# Microbial and metabolomic profiles in correlation with depression and anxiety co-morbidities in diarrhoea-predominant IBS patients

**DOI:** 10.1186/s12866-020-01841-4

**Published:** 2020-06-17

**Authors:** Tong Liu, Xiang Gu, Li-Xiang Li, Ming Li, Bing Li, Xiao Cui, Xiu-li Zuo

**Affiliations:** 1 Department of Gastroenterology, Qilu Hospital, Cheloo College of Medicine, Shandong University, 107 Wenhuaxi Road, Jinan, 250012 Shandong Province China; 2 Laboratory of Translational Gastroenterology, Qilu Hospital, Cheloo College of Medicine, Shandong University, Jinan, China; 3 Robot engineering laboratory for precise diagnosis and therapy of GI tumour, Qilu Hospital, Cheloo College of Medicine, Shandong University, Jinan, China

**Keywords:** Irritable bowel syndrome, Depression, Anxiety, Microbial community, Metabolomics

## Abstract

**Background:**

Psychological co-morbidities in irritable bowel syndrome (IBS) have been widely recognized, whereas less is known regarding the role of gut microbial and host metabolic changes in clinical and psychological symptoms in IBS.

**Results:**

A total of 70 diarrhoea-predominant IBS (IBS-D) patients and 46 healthy controls were enrolled in this study. Stool and urine samples were collected from both groups for 16S rRNA gene sequencing and metabolomic analysis.

The results showed that fecal microbiota in IBS-D featured depleted *Faecalibacterium* (adjusted *P =* 0.034), *Eubacterium rectale* group (adjusted *P =* 0.048), *Subdoligranulum* (adjusted *P =* 0.041) and increased *Prevotella* (adjusted *P =* 0.041). O-ureido-L-serine, 3,4-dihydroxybenzenesulfonic acid and (R)-2-Hydroxyglutarate demonstrated lower urinary concentrations in IBS-D patients. We further built correlation matrices between gut microbe abundance, differentiated metabolite quantities and clinical parameters. *Dialister* manifested negative association with IBS severity (*r* = − 0.285, *P* = 0.017), anxiety (*r* = − 0.347, *P* = 0.003) and depression level (*r* = − 0.308, *P* = 0.010). *Roseburia* was negatively associated with IBS severity (*r* = − 0.298, *P* = 0.012). Twenty metabolites correlated with anxiety or depression levels, including 3,4-dihydroxymandelaldehyde with SAS (*r* = − 0.383, *P* = 0.001), 1-methylxanthine with SDS (*r* = − 0.347, *P* = 0.004) and 1D-chiro-inositol with SAS (*r* = − 0.336, *P* = 0.005). In analysis of microbe-metabolite relationship, 3,4-dihydroxymandelaldehyde and 1-methylxanthine were negatively correlated with relative abundance of *Clostridiumsensu stricto.*

**Conclusions:**

Our findings demonstrated altered microbial and metabolomic profiles associated with clinically and psychological symptoms in IBS-D patients, which may provide insights for further investigations.

## Background

Irritable bowel syndrome (IBS), a common psychosomatic disorder characterized by abdominal pain, bloating and altered bowel habits, is the most commonly diagnosed gastroenterology disorder which affects over 10% of the population globally and the prevalence varies regionally [1, 2]. Despite the relatively good prognosis, IBS affects the quality of life of patients immensely and extensively. Accumulating evidence demonstrated the basic pathophysiology of IBS is disordered brain-gut interaction, frequently accompanied by motility disturbance, visceral hypersensitivity, mild mucosal inflammation, altered gut microbiota, psychiatric disorders and abnormal central nervous system processing [3].

Psychiatric co-morbidities, including higher level of perceived stress, clinically significant depression and/or anxiety, somatization and altered behavioural pattern, are commonly seen in IBS patients and may either predate or generate the intestinal symptoms [4]. It has been reported that up to 94% of IBS patients demonstrate psychiatric disorders, including somatoform disorders, major depression and anxiety [5]. Among multiple pathophysiological mechanisms interpreting the psychiatric manifestations in IBS patients, the commensal gut microbial population has emerged to be potentially significant [6]. Using advanced tools like next generation sequencing, researchers have been allowed to obtain a more thorough overview of gut microbial composition and metabolic processes. Some studies have reported a different microbial pattern in IBS patients and animal models compared to their healthy controls, mainly characterized by altered microbial richness, fluctuated abundance of phyla Firmicutes and Bacteroides. However, existing results are still inconsistent [6, 7].

More recently, gut microbiota has been proposed to interfere with host state via metabolism-regulating ways [8, 9], and efforts have been made to describe a possibly distinct metabolic profile in IBS patients using metabolomic approaches [10, 11]. Additionally, metabolomic tools assist in understanding mechanisms of improving IBS symptoms by treatment like probiotics [12], low fermentable oligo-di-mono-saccharides and polyols (low-FODMAP) diet [13] and non-absorbable antibiotics [14]. Urine is a preferred biomaterial in metabolomic analysis due to its easy accessibility, considerable metabolite concentration and exemption of interfering proteins and lipids compared to serum, faecal extraction and cerebrospinal fluid [15]. However, few studies have combined microbiome and metabolomic data in IBS population, and correlations between microbial, metabolomic changes and psychological alterations in IBS are currently less discussed.

In this study, we hypothesized that diarrhoea-predominant irritable bowel syndrome (IBS-D) patients featured gut microbiota alterations and aberrant metabolism compared with healthy population, which were correlated with IBS bowel symptom and psychological co-morbidity. By detailed analysis of the faecal microbiota and a urinary metabolomic approach, we aimed to characterize those features and further investigate their relationship with IBS-D clinical or psychological status.

## Results

### Characteristics of subjects

Seventy IBS-D patients and 46 healthy controls (HC) were involved in this study. IBS-D duration of patients varied from 6 months to 20 years, with an average ± SD duration of 3.97 ± 5.04 years. No significant differences in gender and age were shown between IBS-D group and HC group. Body mass index (BMI) was well balanced between two groups to decrease its potential effect upon microbial composition and metabolism (Table 1). Bristol stool scale and IBS symptom severity scale (IBS-SSS) reflected significantly severer symptoms in IBS-D group than HC group (Table 1). Several classical psychological scales (see Methods section) were utilised to measure anxiety and depression level of IBS-D patients (Table 1).

**Table 1:** Demographic and clinical data of IBS-D patients and HCs

	IBS-D (*n* = 84)	HC (*n* = 46)	*P* value
Age, Mean (SD)	41.76 (11.57)	38.30 (13.13)	0.139
Sex			
Female	24	21	0.219
Male	46	25	
BMI, Mean (SD)	23.36 (3.49)	23.63 (3.76)	0.700
Bristol scale, Mean (SD)	5.26 (0.70)	3.89 (0.74)	0.000
IBS-SSS, Mean (SD)	286.29 (43.14)	120.22 (44.04)	0.000
Duration, Mean (SD), years	3.97 (5.04)	N^f^	N
SDS^a^	49.89 (9.59)	N	N
SAS^b^	48.81 (9.79)	N	N
HADS^c^	16.57 (6.58)	N	N
HAM-D^d^	13.23 (5.15)	N	N
HAM-A^e^	14.53 (5.86)	N	N

^a^*SDS* Self-reported depression scale, ^b^*SAS* Self-reported anxiety scale, ^c^*HADS* Hospital Anxiety and Depression Scale, ^d^*HAM-D* Hamilton Depression Scale, ^e^*HAM-A* Hamilton Anxiety Scale, ^f^*N* no data

### Characterization of faecal microbiota in IBS-D patients and HCs

**Fig. 1:**
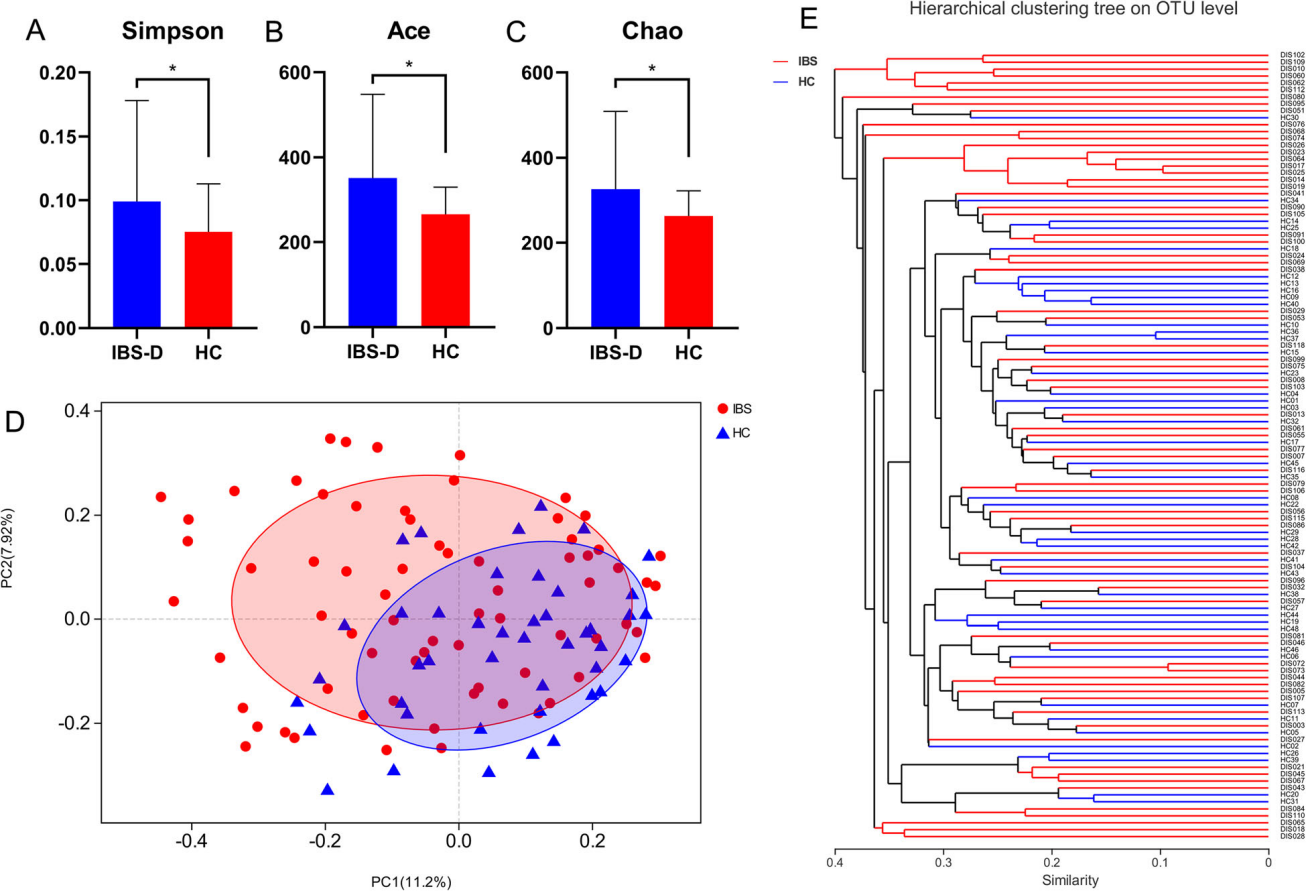
Microbial structures of IBS-D patients and HCs. **a**-**c** Simpson, Ace and Chao index of IBS-D and HC groups. Welch’s test. Means ± SD. **d** PCoA and (**e**) hierarchical clustering of fecal microbiota composition in IBS-D and HC groups based on bray-curtis distance. * *P* < 0.05

Stool samples were collected from 70 IBS-D patients and 44 HCs. After data trimming, we obtained an average of 52,298 (ranging from 32,092 to 72,405) reads per sample. The Good’s coverage reached over 0.997 in both IBS-D and HC group. Simpson index (0.099 ± 0.079 vs. 0.075 ± 0.038, adjusted *P =* 0.041) indicated a higher biodiversity in faeces of HCs (Fig. 1a). Interestingly, Ace (351.17 ± 197.79 vs. 265.74 ± 63.77, adjusted *P =* 0.007) and Chao (326.29 ± 183.19 vs. 263.28 ± 59.05, adjusted *P =* 0.018) reflected that faecal microbial richness in IBS-D patients was higher in comparison of that in HCs (Fig. 1b, c).

Hierarchical clustering and principal co-ordinates analysis (PCoA) based on bray-curtis distances were applied at the level of operational taxonomy unit (OTU) to identify the similarity of microbial community structures between faecal samples from IBS-D patients and HCs (Fig. 1d, e). As shown in Fig. 1e, HC samples were unevenly mixed with IBS-D samples, whereas there were no explicit divisions of two clusters. Similarly, on the PCoA plot, faecal samples from these two groups could not be clearly separated.

**Fig. 2:**
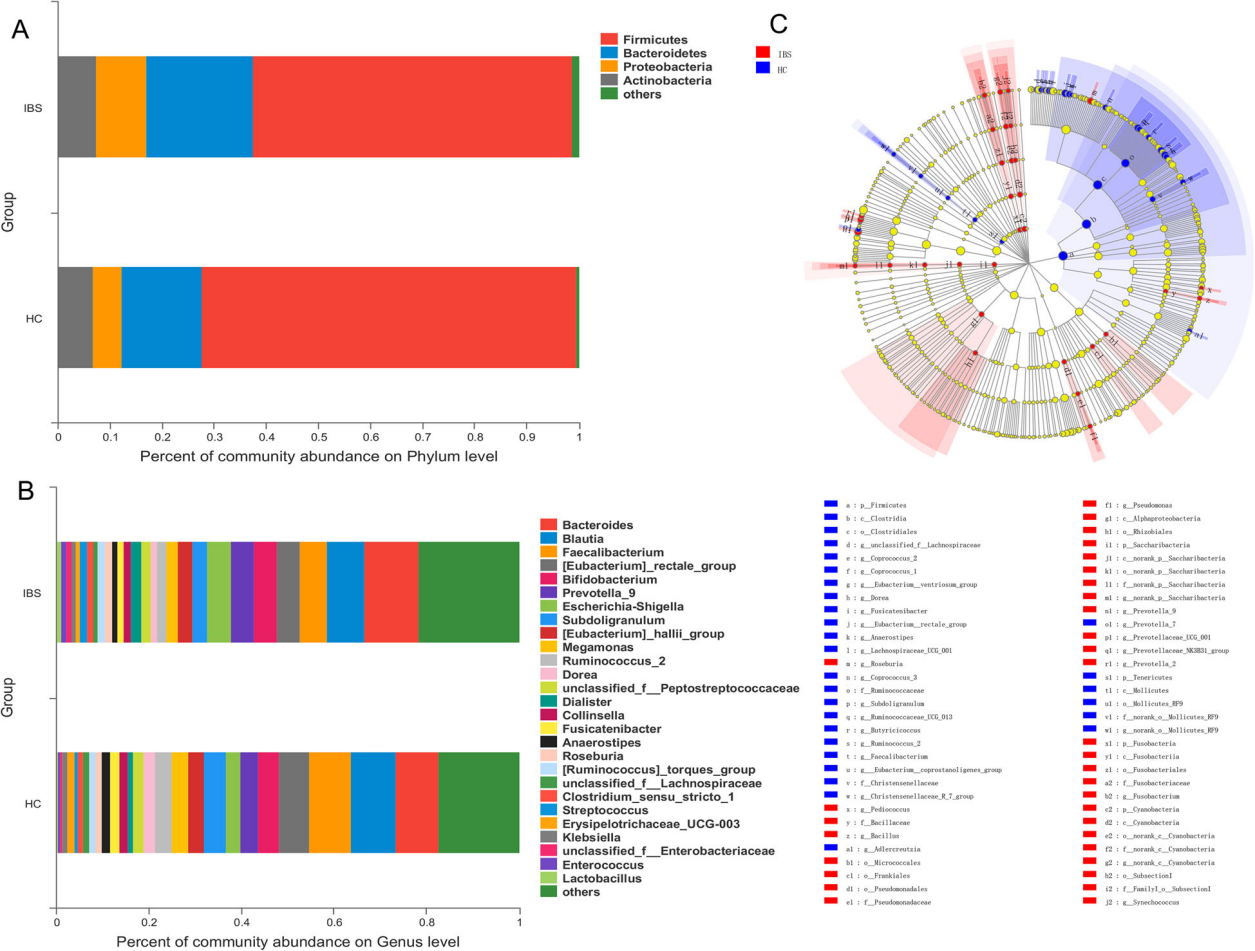
Comparisons of taxonomic abundances in IBS-D and HC groups. Relative taxonomic abundances at the level of (**a**) phylum and (**b**) genus are shown in bar charts. **c** Cladogram from phylum to genus level of LEfSe analysis. Colors indicate taxa enriched in IBS-D or HC population. Diameters of dots are parallel to effect sizes of taxa

Comparisons of faecal microbiota composition between IBS-D and HC group at different taxonomic levels were conducted applying the Mann-Whitney U test. At the phylum level (Fig. 2a), Firmicutes (61.21%), Bacteroidetes (20.52%), Proteobacteria (9.71%) and Actinobacteria (7.32%) were the most abundant phyla of IBS-D group, and in HC group the order was Firmicutes (71.97%), Bacteroidetes (15.42%), Actinobacteria (6.82%) and Proteobacteria (5.48%). The abundance of the most predominant phyla Firmicutes was significantly decreased in IBS-D patients compared with HCs (adjusted *P =* 0.033). Among the first 10 predominant phyla, Cyanobacteria (adjusted *P =* 0.024), Fusobacteria (adjusted *P* < 0.001) and Chloroflexi (adjusted *P* = 0.001) were also significantly higher in faecal samples of IBS-D patients though their abundances were lower than 1% in both groups. *Faecalibacterium* (6.02% ± 6.48% vs. 9.14% ± 6.33%, adjusted *P =* 0.034), *Eubacterium rectale* group (4.87% ± 6.83% vs. 6.48% ± 5.70%, adjusted *P =* 0.048) and *Subdoligranulum* (3.20% ± 4.78% vs. 4.87% ± 4.55%, adjusted *P =* 0.041) were genera of higher abundances in stool samples of HC group, while *Prevotella* 9 (4.84% ± 11.39% vs. 3.75% ± 8.52%, adjusted *P =* 0.041) was increased in IBS-D group. *Bacteroides* (adjusted *P =* 0.826) and *Blautia* (adjusted *P =* 0.137) were the most predominant genera in IBS-D and HC group (Fig. 2b), respectively. Yet, the differences of both between 2 groups didn’t reach significance after false discovery rate (FDR) correction. Cladogram from phylum to genus level using linear discriminant analysis effect size (LEfSe) demonstrated specific faecal microbial taxa associated with IBS-D or health (Fig. 2c). Most taxa of high linear discriminant analysis (LDA) scores belonged to the phylum Firmicutes, which had the potential to be identifying biomarkers for IBS-D or HC (Fig. S1).

### Associations between clinical and psychological characteristics and differential microbiota composition in IBS-D

**Fig. 3:**
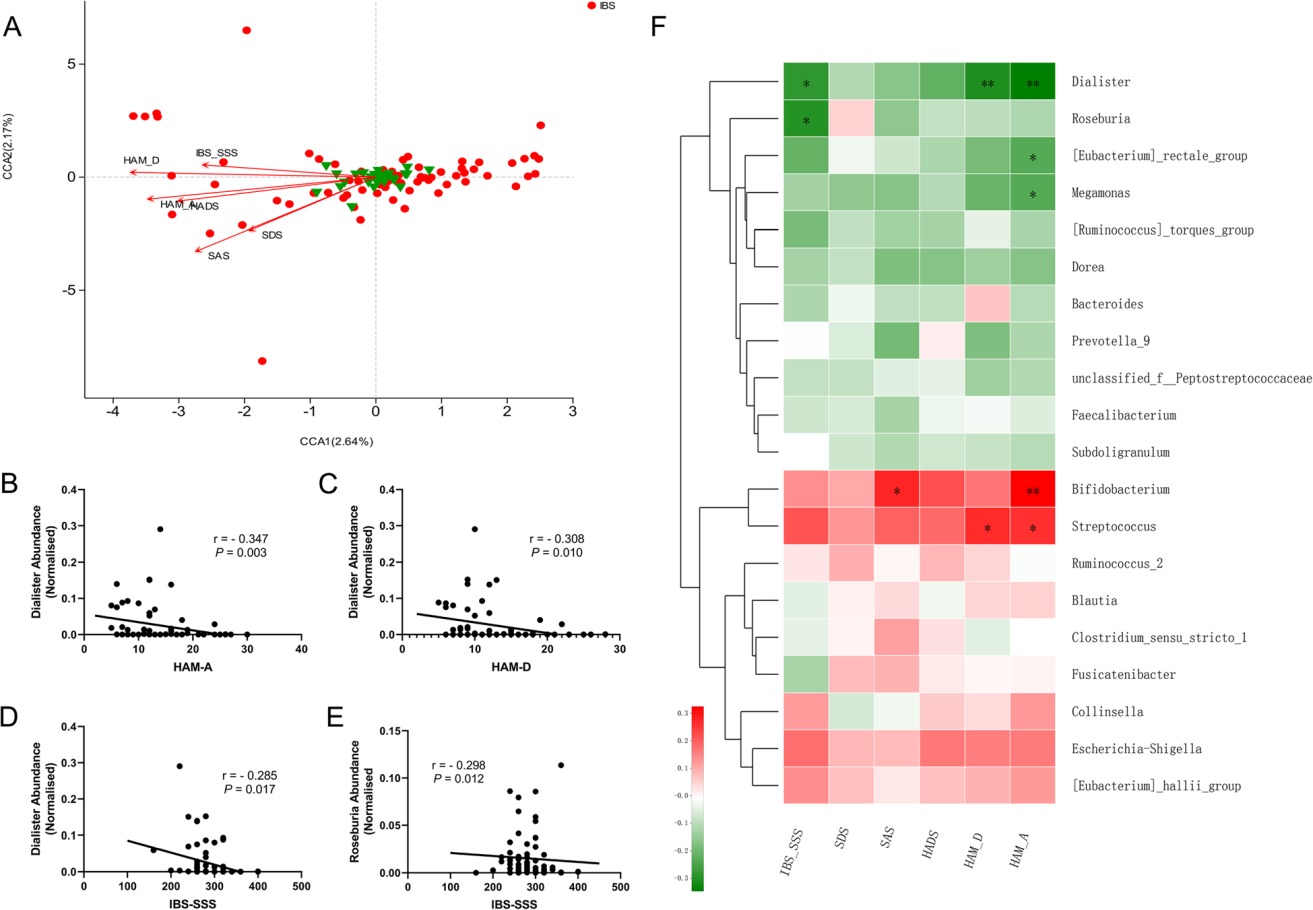
Correlations between clinical characteristics and faecal microbial abundances in IBS-D patients. **a** Canonical correspondence analysis (CCA) plot in clinical scales (arrows), genera (green triangles) and faecal samples (red dots). **b**-**e** Scatter plot Spearman’s correlation between featured genera and scales. **f** Heatmap of correlations between abundances of genera and clinical scales. Positive and negative correlations are shown in red and green blocks, respectively. * *P* < 0.05, ** *P* < 0.01

We included six variables regarding clinical symptoms and psychological abnormalities (IBS-SSS, Self-reported Anxiety and Depression Scales (SAS / SDS), Hospital Anxiety and Depression Scale (HADS), Hamilton Anxiety and Depression Scales (HAM-A / HAM-D)) that may be related to microbial signature in 70 IBS-D patients. All variables were included after variance inflation factor analysis at a threshold of 10 to rule out redundant parameters. Canonical correspondence analysis (CCA) demonstrated the extent to which those factors shaped the microbial community in IBS-D (Fig. 3a, Table S1). All six factor scores significantly correlated with microbial structure (adjusted *P* ≤ 0.001).

Furtherly, we examined associations between composition of faecal microbiota and those clinical metadata using Spearman’s correlation coefficient. Significant correlations were found between relative abundances of genus *Roseburia* and IBS severity (*r* = − 0.298, *P* = 0.012), *Dialister* and IBS severity (*r* = − 0.285, *P* = 0.017), *Dialister* and HAM-D (*r* = − 0.308, *P* = 0.010), *Dialister* and HAM-A (*r* = − 0.347, *P* = 0.003), *Bifidobacterium* and SAS (*r* = 0.276, *P* = 0.021), *Bifidobacterium* and HAM-A (*r* = 0.325, *P* = 0.006), *Megamonas* and HAM-A (*r* = − 0.235, *P* = 0.049), *Streptococcus* and HAM-D (*r* = 0.269, *P* = 0.024), *Streptococcus* and HAM-A (*r* = 0.262, *P* = 0.029) (Fig. 3b-f). We then employed the multivariate statistical approach to relate psychological scale parameters to microbial abundances for which age, gender and IBS severity were adjusted considering their confounding effect upon gut microbial structure. In the adjusted multivariate model, we detected significant negative associations between *Dialister* quantity with SAS and SDS as well as *Clostridiumsensu stricto* with HADS and HAM-D. The relative abundance of *Bifidobacterium* was positively associated with anxiety scores in HAM-A and SAS, in parallel with Spearman’s correlation results (Table S2).

### Differentiated urinary metabolic profiles of IBS-D patients and HCs

**Fig. 4:**
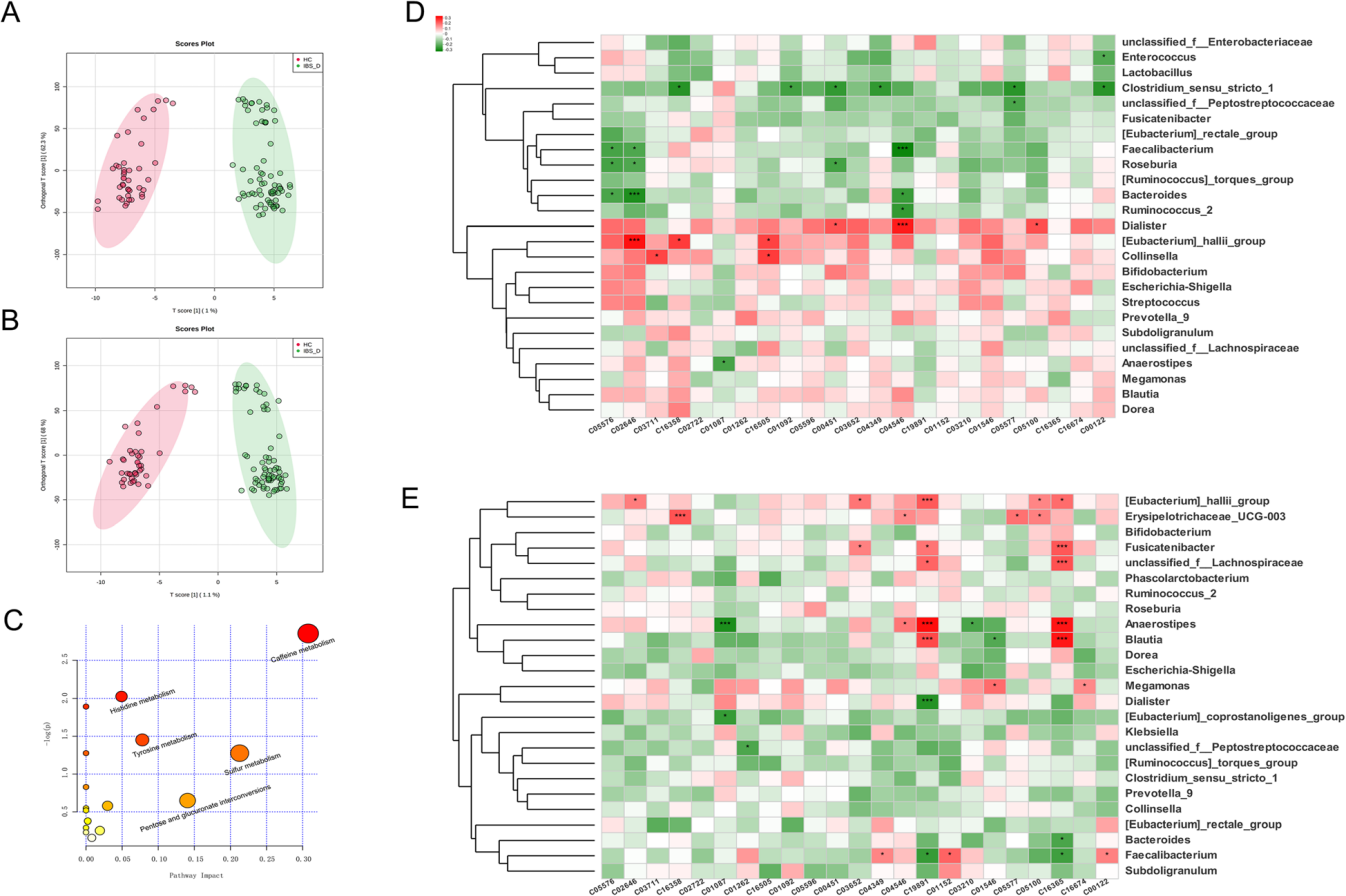
Urinary metabolomics in IBS-D and HCs and correlations between faecal microbial abundances and discriminative metabolites. OPLS-DA scores plots for IBS-D (green) and HC (blue) samples in (**a**) ESI^−^ and (**b**) ESI^+^ modes of UHPLC-MS/MS. **c** Bubble plot of metabolic pathways associated with urinary metabolites differentiating IBS-D and HC group. Horizontal axis and bubble sizes indicate impact of pathways in topology analysis. Vertical axis and bubble color indicate - lg (*P* value) of pathways in enrichment analysis. **d**-**e** Heatmap of correlations between abundances of genera and metabolites associated with changed clinical scales in IBS-D (**d**) and HCs (**e**). Positive and negative correlations are shown in red and green blocks, respectively. Spearman’s correlation coefficients. * *P* < 0.05, ** *P* < 0.01

Ultra-high performance liquid chromatography tandem mass spectrometry (UHPLC-MS/MS) harvested urinary metabolomic data from 70 IBS-D and 42 HC samples. Orthogonal partial least squares-discrimination analysis (OPLS-DA) was employed to explore the differentiated metabolites between two groups (Figs. 4a, b). The robustness of OPLS-DA models was verified by 7-fold cross-validation (Q^2^ = 0.544 for ESI^−^ mode and Q^2^ = 0.511 for ESI^+^ mode) and 200 times of permutation tests were utilised to exclude overfitting models (Q^2^ intercept < 0 for both ESI^−^ and ESI^+^ modes). After removal of duplicates, a total of 114 metabolites emerged to be significantly different between IBS-D group and HC group (Variable importance in the projection (VIP) > 1.00, adjusted *P <* 0.05), among which 50 metabolites were annotated with Kyoto Encyclopedia of Genes and Genomes (KEGG) compound IDs (Table S3). Differentiated metabolites with the highest VIPs are O-ureido-L-serine (VIP = 6.27, HC/IBS-D fold change (FC) = 2.23), 3,4-dihydroxybenzenesulfonic acid (VIP = 6.24, FC = 2.60), (R)-2-hydroxyglutarate (VIP = 5.67, FC = 3.16), (2R_3S)-2_3-dimethylmalate (VIP = 5.35, FC = 0.07). After mapping differentiated metabolites into KEGG metabolic pathways and conducting enrichment analysis and topology analysis, caffeine metabolism pathway exhibited highest impact value of 0.31. However, no metabolism pathways demonstrated significant distinction between IBS-D and HCs (Fig. 4c, Table S4).

### Urinary metabolites associated with psychological parameters and microbiota composition of IBS-D patients

In the aim of combining metabolomic outcomes and clinical characteristics of IBS-D patients, we calculated the correlations between differentiated urinary metabolite quantities and IBS clinical parameters (Table 2). Among the metabolites which were negatively associated with depression or anxiety level, 3,4-dihydroxymandelaldehyde manifested best correlation with SAS (*r* = − 0.383, *P* = 0.001), followed by 1-methylxanthine (with SDS, *r* = − 0.347, *P* = 0.004), 4-hydroxyphenylacetylglycine (with SAS, *r* = − 0.337, *P* = 0.005) and 1D-chiro-inositol (with SAS, *r* = − 0.336, *P* = 0.005). Some metabolites also manifested positive or negative correlations with IBS symptom severity, including 2-furoate (*r* = 0.424, *P* < 0.001) and anserine (*r* = − 0.296, *P* = 0.002), etc. Interestingly, 1-methylhistidine showed negative associations with not only symptom severity (*r* = − 0.260, *P* = 0.007) but SAS (*r* = − 0.263, *P* = 0.007) and SDS (*r* = − 0.250, *P* = 0.031). When age, gender and IBS symptom severity were adjusted for, significant relationships between urinary quantities of annotated metabolites and psychological outcomes were shown in the multivariate linear regression models (Table S5).

**Table 2:** Associations between clinical characteristics and differential urinary metabolites in IBS-D patients

KEGG ID	Metabolite Name	Related Parameter	Spearman’s Coefficient	*P value*
C05576	3_4-Dihydroxyphenylethyleneglycol	IBS-SSS^a^	0.216	*0.026*
C02722	n-Hexanamide	IBS-SSS	0.208	*0.032*
C01546	2-Furoate	IBS-SSS	0.424	*0.000*
C16505	(10S)-Juvenile hormone III diol	IBS-SSS	0.366	*0.000*
C16674	formyl-isoglutamine	IBS-SSS	0.329	*0.001*
C01262	Anserine	IBS-SSS	- 0.296	*0.002*
C01152	1-Methylhistidine	IBS-SSS	- 0.260	*0.007*
C03652	(2R_3S)-2_3-Dimethylmalate	IBS-SSS	0.250	*0.009*
C16365	5-Acetylamino-6-formylamino-3-methyluracil	IBS-SSS	- 0.236	*0.014*
C19891	1D-chiro-inositol	IBS-SSS	- 0.230	*0.017*
C00122	Fumaricacid	IBS-SSS	- 0.218	*0.024*
C00451	(1R_2S)-1-Hydroxypropane-1_2_3-tricarboxylate	IBS-SSS	0.196	*0.043*
C03210	2_2’-Iminodipropanoate	IBS-SSS	0.192	*0.047*
C16358	1-Methylxanthine	SAS^b^	- 0.286	*0.019*
C05577	3_4-Dihydroxymandelaldehyde	SAS	- 0.383	*0.001*
C05596	4-Hydroxyphenylacetylglycine	SAS	- 0.337	*0.005*
C19891	1D-chiro-inositol	SAS	- 0.336	*0.005*
C16674	formyl-isoglutamine	SAS	- 0.306	*0.012*
C04349	(4S)-4_6-Dihydroxy-2_5-dioxohexanoate	SAS	- 0.294	*0.016*
C00122	Fumaricacid	SAS	- 0.292	*0.017*
C05100	3-Ureidoisobutyrate	SAS	- 0.281	*0.021*
C01152	1-Methylhistidine	SAS	- 0.263	*0.031*
C16358	1-Methylxanthine	SDS^c^	- 0.347	*0.004*
C03711	N-Methylphenylethanolamine	SDS	- 0.266	*0.030*
C02646	4-Coumarylalcohol	SDS	- 0.245	*0.046*
C03652	(2R_3S)-2_3-Dimethylmalate	SDS	- 0.268	*0.028*
C05577	3_4-Dihydroxymandelaldehyde	SDS	- 0.267	*0.029*
C01087	(R)-2-Hydroxyglutarate	SDS	- 0.266	*0.030*
C01092	8-Amino-7-oxononanoate	SDS	- 0.265	*0.030*
C01152	1-Methylhistidine	SDS	- 0.250	*0.042*
C05596	4-Hydroxyphenylacetylglycine	SDS	- 0.242	*0.049*
C04546	(R)-3-((R)-3-Hydroxybutanoyloxy)butanoate	SDS	- 0.242	*0.049*
C01262	Anserine	HAM-A^d^	- 0.253	*0.039*

^a^*IBS-SSS* IBS symptom severity scale, ^b^*SAS* Self-reported anxiety scale, ^c^*SDS* Self-reported depression scale, ^d^*HAM-A* Hamilton anxiety scale

To further investigate the relationship between urinary metabolites and faecal microbiome, correlation matrices based on spearman’s coefficient were constructed for IBS-D and HC group, respectively (Fig. 4d, e). For IBS-D patients, both 3,4-dihydroxymandelaldehyde and 1-methylxanthine were negatively correlated with the relative abundance of *Clostridiumsensu stricto.* Significant relationships were also found in positive correlation between (R)-3-((R)-3-hydroxybutanoyloxy) butanoate and *Dialister* (*r* = 0.312, *P* = 0.008), and negative association of (R)-3-((R)-3-hydroxybutanoyloxy) butanoate and *Faecalibacterium* (*r* = − 0.335, *P* = 0.005), etc. However, none of these significant relationships was demonstrated in the correlation matrix of HC population, suggesting the distinction of microbe-related metabolites between two groups. Quantitative data of metabolites and gut microbial genera with abundances > 1% were also used to construct correlation networks to visualize a full-scale view of the microbiome-metabolite relationship via Cytoscape platform (Fig. S2).

## Discussion

In this study, we examined the relationship between clinical and psychological abnormalities, faecal microbiome and urinary metabolic profile in IBS-D patients, potentially supporting further mechanism researches. To our knowledge, this is the first study to combine faecal microbiome and urinary metabolome data with psychological symptoms in IBS to date.

Previous studies have determined that IBS patients featured gut microbiota alterations, which contributed to IBS pathophysiology [16]. However, results concerning microbial diversity and composition were divergent, which might arise from the heterogeneity of IBS. Our current study compared the faecal microbiota from IBS-D and healthy groups. Simpson index demonstrated decreased faecal biodiversity, whereas Ace and Chao indices showed higher microbial richness in IBS-D group, supported by human and animal researches [17–19]. Analysis of similarity (ANOSIM), PCoA and hierarchical clustering analysis based on Bray-Curtis distance showed no significant difference of β-diversity between groups. Inconsistently, some studies conducted in Chinese or western population also revealed decreased or unchanged microbial α-diversity and richness in IBS-D [8, 18, 20, 21]. Interestingly, “normal-like” microbiota in IBS subgroup, which might result in undetected selection bias and inconsistency of overall compositional results, was suggested by accumulating reports [22, 23]. From the perspective of bacterial taxonomic alterations, most high-throughput data showed a decreased abundance of phyla Firmicutes and increased Bacteroidetes in IBS microbes in agreement with our results [21, 24]. Vast studies demonstrated that *Bifidobacterium* and *Lactobacillus* were less abundant in IBS-D, whereas *Clostridium*, *Escherichia coli* and *Enterobacter* were often increased [19, 20, 24–26]. *Faecalibacterium* (or *F. prausnitzii*) abundance in IBS increased in some studies [27], but constant or decreased in others [24]. However, a recent meta-analysis that included 17 studies argued that species-specific alterations of gut microbiota were different between IBS patients from China and other regions [25]. Differentiation of microbial composition also existed among different IBS sub-categories (especially between IBS-D and constipation-predominant IBS), probably due to distinct manifestations of IBS bowel motility [24, 27, 28]. Here we found *Faecalibacterium*, *Subdoligranulum* and *Eubacterium rectale* group, all capable of producing butyrate [29], were depleted in IBS-D faecal samples. Researches have demonstrated that short chain fatty acids (SCFAs), of which butyrate is more extensively studied [30], are intestinal fermentative products that play a critical role in protecting intestinal mucosa, regulating innate and systematic immune response and improving gastrointestinal motility [31]. For IBS patients, SCFA-producing microorganisms such as *Roseburia, Faecalibacterium*, *Eubacterium* and Ruminococcaceae family and were reported depleted [8, 32–34]. Interestingly, low-FODMAP diet, which effectively improved IBS bloating and distension symptoms [13, 35], could also result in reduced intestinal butyrate abundance and butyrate-producing bacteria [13, 36–38]. These findings suggested the complex interplay between diets, IBS microbiome and symptoms, and warranted further diet-management investigations. It’s also interesting that *Prevotella* abundance increased in IBS-D group. Previous studies have shown that *Prevotella* was associated with immunological imbalance and localised or systemic diseases, probably through promoting mucosal Th17 immune responses [39–42]. A recent report revealed that *Prevotella* was depleted in faeces from IBS patients and post-infectious IBS (PI-IBS) mice model, and might be positively associated with a high risk of development of IBS hypersensitivity [20]. Considering the theory that IBS embodies low-grade and chronic inflammation [43], further investigations on the role of *Prevotella* in IBS and other gut inflammatory alterations are of interest.

Metabolomics has been broadly acknowledged to be closely associated with phenotypes in health and diseases [44]. Earlier targeted metabolomics studies primarily focused on several categories of metabolites, including SCFAs, amino acids, bile acids, etc. [26, 45]. In contrast, non-targeted metabolomics approaches under development collected more detailed and global metabolic profiling data concerning metabolic homeostasis and aberrance [14, 19, 46, 47]. Growing data had demonstrated that metabolism was disturbed in IBS of animal model [48] or patients [14], whereas detected metabolites were in divergence among studies. Faecal or serum samples used to be largely utilised in metabolomic studies of IBS and other diseases. In comparison, urine was much easier to obtain at an outpatient clinic, considered sterile, and free from bio-macromolecules [15], therefore could be apt for metabolomic analysis. Recent research on proton nuclear magnetic resonance (^1^HNMR)-based urinary metabolomics recruited 16 healthy volunteers and 8 IBS-D patients and indicated lower concentration of indoxyl-sulphate and increase of methylamine and taurine in IBS-D [49]. Another study recruited 76 IBS participants at different time points and detected 10 metabolites consistently elevated in IBS, including a series of hydroxylysine metabolites, amino acids and SCFA derivatives [50]. Here our data harvested 114 differentiated metabolites (50 with KEGG ID annotation) between IBS-D patients and HCs, demonstrating higher sensitivity of UHPLC-MS/MS-based metabolomics (relevant studies listed in Table S6). Among the annotated metabolites, some organic acids (3,4-dihydroxybenzenesulfonic acid, (R)-2-hydroxyglutarate, 2,3-dimethylmalate, (1R,2S)-1-hydroxypropane-1,2,3-tricarboxylate, etc.) and N-phenylacetylglutamine featured highest VIPs telling IBS-D from HC samples. However, no statistically significant difference was observed in the pathway enrichment analysis between IBS-D and HCs in our study, in contrast to previous reports that alanine, aspartate, and glutamate metabolism, amino sugar and nucleotide sugar metabolism were disturbed in IBS [14].

Though accumulating researches have discussed microbial and metabolic alterations in IBS, few focused on their relationships with IBS symptoms, especially psychological abnormalities. Herein, we analysed correlations between clinical data and faecal microbial in a comparatively large population of IBS patients. *Dialister* abundance was negatively correlated with IBS symptom severity, anxiety and depression level. A study of diet therapy demonstrated that more significant improvements in systematic inflammatory levels were related to higher proportions of *Dialister* [51]. Previous works also suggested that *Dialister* was deficient in depression (corrected for antidepressant intake) [52] and some paediatric diseases, including food sensitization [53], self-immune diseases [54] and autism spectrum disorders [55]. Modest positive associations took places between anxiety status and *Bifidobacterium* or *Streptococcus*. Population with chronic stress demonstrated increased gut *Streptococcus*, which could be attenuated by probiotic *Lactobacillus gasseri.* Inconsistently with our results, probiotic preparations of *Bifidobacterium* improved mental state in previous researches [12, 56]. We also found that relative abundance of *Roseburia* was negatively correlated with IBS severity. *Roseburia* produces SCFAs, mainly butyrate, in the human colon [57]. Studies have reported that patients with constipated-predominated IBS [32] and ulcerative colitis also demonstrated reduced *Roseburia* [58]*.* Treatment of *R. hominis* seemed to be anti-inflammatory in an immune-regulating manner [58–60]. Our previous study [61] has shown the effectiveness of *Clostridium butyricum*, which is also a butyrate producer in genus *Clostridiumsensu stricto*, in improving IBS-D symptoms and a similar potential of *Roseburia* in IBS may be worth further investigation. Considering the potential heterogeneity of microbiota driven by age, sex [62] and symptom severity [63], we also built multivariate linear regression models with adjustment of those parameters. Interestingly, in addition to previously detected genera, *Clostridiumsensu stricto* was negatively associated with depression level in the final model.

Moreover, in analysing correlations between clinical data and differentiated urinary metabolites, some metabolites were found positively or negatively associated with IBS severity, anxiety or depression level. A large proportion of those metabolites mainly involved in amino acid metabolism, including tyrosine metabolism (3,4-dihydroxymandelaldehyde, 4-hydroxyphenylacetylglycine), histidine metabolism (1-methylhistidine, anserine) and beta-alanine metabolism (anserine). Alterations in amino acid metabolism were also reported in IBD, celiac disease and intestinal malignancies [64–67]. In our results, the anxiety level of IBS-D patients was negatively correlated with urinary 1D-chiro-inositol, an enantiomer of myo-inositol. Concentration of myo-inositol in central nervous system had been reported to be altered in autism [68, 69] and depression of adolescents [70]. Additionally, magnetic resonance spectroscopy - derived brain myo-inositol concentration could be positively predicted by both intestinal *Bacteroides* and *Clostridium XIVb*, another suggestion that microbe affects mental status via metabolic regulation [71]. Negative relationships existed between 1-methylxanthine and SDS as well as between 3,4-dihydroxymandelaldehyde and SAS. It was reported that caffeine helped attenuate depression and anxiety behaviour in a dose-dependent manner [72], and it was not surprising that its metabolite, 1-methylxanthine, was a negative indicator for depression scale. IBS-D patients had a reduced 3,4-dihydroxymandelaldehyde level in urine observed in our data. In vivo, it was a monoamine oxidase aldehyde metabolite of norepinephrine. Previous research suggested that increased urinary excretion of norepinephrine metabolites (including 3,4-dihydroxymandelaldehyde and vanillylmandelic acid) was predictive of improvement in depressive and anxiety states [73].

Our study combined clinical, microbial and metabolomic data and suggested a possible correlation network among them, as previous evidence suggested the function of microbiota in regulating emotions was potentially in a metabolism modifying manner [74, 75]. For instance, the anxiety-like phenotype was induced in recipient mice by transplantation of IBS-D faeces, in parallel with changed serum metabolism profiles [75]. In the current study, correlation analysis hinted that two mental state-related metabolites, 1-methylxanthine and 3,4-dihydroxymandelaldehyde, were in negative correlation with the relative abundance of *Clostridiumsensu stricto,* suggesting this genus might relate with worsening of psychological abnormality. Social stressor induced an increased abundance of *Clostridium* in mice [76], and an elevated *Clostridium* and butyrate in faeces were seen in IBS patients [77]. *Clostridium*-produced butyrate, though commonly accepted as an anti-inflammatory and protective factor in IBS, may also promote intestinal hypersensitivity seen in animal and human [61, 78, 79]. The potential mechanisms of butyrate aggravated bowel hypersensitivity were reported to involve peptidergic C-fibres, spinal cord plasticity, acid-sensing ion channels 1A (ASIC1A) and modulation of 5-hydroxytryptamine (5-HT or serotonin) release [80]. Also, isobutyrate, another metabolite of *Clostridium*, was found correlated significantly with decreased anxiolytic and anti-depressive effects of prebiotics [81]. In our previous study, *Clostridiumsensu stricto* was depleted in IBS patients with improved quality of life after interfering with probiotics, suggesting its role of predicting post-treatment outcome [61]. Positive correlation between (R)-3-((R)-3-hydroxybutanoyloxy) butanoate and *Dialister* was also in alignment with the finding that *Dialister* indicated a lower depression level. However, organisms related to metabolism disturbances shown in correlation matrix were not in proper alignment with those differed between HCs and IBS-D patients, and the specific metabolism-regulating mechanisms of intestinal flora impacting on IBS emotions and symptoms warrant further in vivo researches.

We acknowledge that there are several limitations in our study. Firstly, the collected microbiota, metabolomic data and clinical metadata lacked dietary information and follow-up investigation, potentially confounding the present results. Secondly, the sample size of our single-centre exploratory study was still small, which limited the generalization of the conclusions. However, the present sample size had a sufficient statistical power for comparing microbiome between IBS-D and HC groups (see in Methods). Selection bias of study subjects might exist, though main confounding factors including gender and age were controlled in the multivariate linear regression analyses. It’s also emphasized that further mechanism studies are required to develop clinical tools concerning microbe- or metabolism-regulated mental health especially in IBS.

## Conclusion

In the current study, we identified specific urinary metabolites and faecal microbes which had the potential to be biomarkers for differentiating IBS patients and healthy controls, though inconsistency with former studies and limited sample size warranted further validation. We also demonstrated that specific microbial genera and metabolites were correlated with not only IBS bowel symptoms but also psychological status including depression and anxiety, suggesting microbiome- or metabolism-oriented diagnostic tools or treatments in IBS would be worth further investigation. Modest to strong associations were observed between metabolites and microbes, corroborating the theory that gut microbes were likely to interplay with physical and mental status via interfering with human metabolism.

## Methods

### Participants

IBS-D patients fulfilling Rome IV criteria were recruited from Department of Gastroenterology at Qilu Hospital of Shandong University between September 2016 and May 2018. The aim of enrolling only IBS-D patients was to eliminate the potential difference between IBS sub-phenotypes and focus on the most prevalent one in China. Patients suspected of PI-IBS were also excluded due to the heterogeneity in pathophysiology and probable bias in microbial composition. Meanwhile, HC subjects were recruited through advertisement and were defined as individuals with colonoscopy of no negative results and without current or previous gastroenterological symptoms nor history of chronic diseases. None of enrolled subjects were on medication including antibiotics, probiotics, prebiotics, laxatives, non-steroidal anti-inflammatory drugs, proton pump inhibitors, steroids, mast cell stabilizers, histamine antagonists, immunosuppressant agents and depressants. No patients nor control subjects were diagnosed with organic diseases including inflammatory bowel disease, coeliac disease, anaphylactic diseases and clinical psychiatric disorders. No patients nor control subjects had undergone gallbladder surgery ever or other major surgeries within half a year.

Basic information and IBS-SSS [82] and Bristol stool scale were provided by participants of both groups. SAS, SDS [83], HADS [84] and HAM-A, HAM-D [85] were utilised to measure anxiety and depression level of IBS-D patients by experienced physicians.

### Sample collection and storage

Stool samples and urine samples were collected from IBS-D patients and HCs and kept in empty sterile tubes. All the stool and urine samples were stored at − 80 °C as soon as possible (delay in transfer less than 5 min). Minimal mass required for stool sample per tube was 1.0 g and minimal volume for urine sample per tube was 5.0 ml. All samples were collectively transferred to Majorbio (Shanghai, China) for further analysis.

### 16S ribosomal RNA (rRNA) gene sequencing

Microbial DNA was extracted from fecal samples from IBS-D patients and HCs using the E.Z.N.A.® Soil DNA Kit (Omega Bio-tek) according to kit protocols. DNA concentration and purity were assessed by Nanodrop (Thermo Scientific) and DNA quality was determined by 1% agarose gel electrophoresis. The V3-V4 regions of the bacteria 16S rRNA gene were amplified by PCR using the primers: 338F 5′-ACTCCTACGGGAGGCAGCA-3′ and 806R 5′-GGACTACHVGGGTWTCTAAT-3′. The PCR products were then extracted from 2% agarose gels and further purified by using the AxyPrep DNA Gel Extraction Kit (Axygen Biosciences) and quantified by QuantiFluor™ -ST (Promega) according to protocols. The purified DNA amplicons were then conducted by Majorbio (Shanghai, China) and were sequenced on Illumina MiSeq platform according to the standard protocols.

Harvested reads were stored and mostly analysed on Majorbio i-Sanger Cloud Platform (http://www.i-sanger.com). Reads were firstly trimmed and clustering of similar sequences into OTU was processed with a 97% sequence identity using Usearch (version 7.0 http://drive5.com/uparse/). Dereplication, discard of singletons (no less than 5 reads in at least 5 samples) and rarefaction based on the minimal number of reads among samples were conducted before analysis. Ace index and Chao index were calculated to evaluate microbial richness in each sample, and Shannon index, Simpson index for alpha-diversity. Sequences from all samples were classified by Bayesian algorithm using the Ribosomal Database Project (RDP) classifier. Hierarchical clustering and PCoA on a level of all OTUs were performed based on bray-curtis distances. ANOSIM based on Bray-Curtis distance and 999 times of permutation tests was utilised to analyse structural difference between IBS-D and HC groups via R. LEfSe calculation was conducted from phylum to genus level between two groups at the LDA score threshold of 2.5.

### UHPLC-MS/MS

Metabolism analysis was performed using a Ultimate 3000 ultra-high performance liquid chromatography system (Dionex, Sunnyvale, CA, United States) united with Q Exactive™ Hybrid Quadrupole-Orbitrap Mass Spectrometer equipped with an electrospray isolation (ESI) source consisting of both positive and negative ion modes (Thermo Fisher Scientific, Waltham, MA, United States). 100 μl urine per sample was mixed with 100 μl pure water containing internal standard, centrifuged at 13000 rpm, 4 °C for 10 min. 5 μl of supernatant per sample was transferred to vial for UHPLC-MS/MS analysis. LC conditions were as follows: column, Acquity UPLC HSS T3 column (100 mm × 2.1 mm, 1.7 μm, Waters, USA); temperature, 40 °C; flow rate, 0.35 ml/min; separation was achieved by using the following gradient: 0–1.0 min, 5% B; 1.0–9.0 min, 5 ~ 100% B; 9.0–12.0 min, 1000% B, 12.0–15.0 min, 5% B, where B is acetonitrile (0.1% (v/v) formic acid) and A is aqueous formic acid (0.1% (v/v) formic acid). The Q Exactive™ mass spectrometer was used for its ability to acquire MS/MS spectra on information-dependent acquisition (IDA) mode in the control of the acquisition software (Xcalibur 4.0.27, Thermo). In this mode, the acquisition software continuously evaluates the full scan MS spectrum. The ESI source conditions were set as following: sheath gas flow rate as 45 Arb, Aux gas flow rate as 15Arb, capillary temperature 400 °C, full MS resolution as 70,000, MS/MS resolution as 17,500, collision energy as 20/40/60 eV in NCE mode, spray Voltage as 4.0 kV (positive) or − 3.6 kV (negative), respectively. The raw data were converted to the mzML format using ProteoWizard and processed with an in-house program, which was developed using R and based on XCMS, for peak detection, extraction, alignment, and integration. Then an in-house MS2 database (BiozeronDB) was applied in metabolite annotation. The cut off value for annotation was set at 0.3. To guarantee the reliability of metabolomic results, quality control (QC) samples were obtained as mixture of aliquots from all urinary samples, and were evenly distributed at intervals of every 10 tested samples throughout the analytical array. Extracted data raw data were trimmed using Compound Discoverer 2.1 (Thermo Fisher Scientific, Waltham, MA, United States) and imported into SIMCA (version 14.0, Umetrics, Sweden) for PCA and OPLS-DA. Comparisons of metabolite concentrations between IBS-D and HC groups were performed using student’s t-test. Differentiated metabolites were harvested with criteria of adjusted *P* < 0.05, VIP > 1.0 and then annotated by KEGG pathway database. Enrichment analysis and topological analysis were performed on MetaboAnalyst platform (https://www.metaboanalyst.ca/MetaboAnalyst/faces/ModuleView.xhtml).

### Statistical analyses

Pearson’s Chi-squared test was used in proportion data and Welch’s t-test in data with normal distribution. For microbiota analysis, Welch’s t-test was used in diversity index calculation. Different taxa between two groups was determined by Mann-Whitney U test. CCA was utilised to analyse the relationship between gut microbial structure and clinical metadata. Redundant variables were gradually removed until all of included variables featured a variance inflation factor below 10. In CCA model, contribution of each environmental factor (clinical metadata) to microbial structure modification was calculated based on 999 Monte Carlo permutations followed by Benjamini-Hochberg correction using envfit function of R. We used multivariate linear regression models to assess the association between psychological scale parameters and microbial abundances/metabolite quantities. We tested the 20 most abundant genera or 50 KEGG ID-annotated metabolites in IBS-D as potential covariances. We built independent models for each genus with age, gender, and IBS severity (IBS-SSS < 300 as mild, ≥ 300 as severe) forced in. Genera with a *P* value under 0.05 were selected into the final multivariate linear regression models. Spearman correlation coefficients were calculated for relationships between environmental factors, taxonomic abundances and metabolomic data. Correlation network between metabolomic and microbial data was plotted (correlation edges with spearman’s coefficient > 0.3 / < − 0.3 and *P* < 0.05 were shown) and visualised by the open-source Cytoscape v.3.2.2. We conducted post hoc power calculation for the comparison of differential OTU abundances between IBS and HC groups (stricter than simply compare alpha- and beta-diversity) using a published Shiny-based microbiome power calculator (http://fedematt.shinyapps.io/shinyMB/) [86]. The Monte Carlo simulation and Wilcoxon–Mann–Whitney test based calculator demonstrated that our current samples (70 IBS and 44 HC) had sufficient power (0.81) to detect a 30% fluctuation in at least 4 most abundant OTUs between 2 groups. All *P* values were two-sided. FDR following Benjamini-Hochberg procedure was applied under multiple-testing circumstances. *P* or adjusted *P* values below 0.05 were considered statistically significant. Statistical analyses were performed using SPSS version 23.0 (SPSS, Inc., Chicago, IL, United States) and R v.3.6.2.

## Supplementary information

**Additional file 1.**

**Additional file 2.**

**Additional file 3.**

**Additional file 4.**

**Additional file 5.**

## Data Availability

The dataset of 16S rRNA gene sequencing supporting the conclusions of this article is available in the National Center Biotechnology Information repository with accession code PRJNA544721. Urinary metabolomic raw data has been deposited to Figshare repository with the assigned DOI: 10.6084/m9.figshare.8218889.

## Abbreviations

IBS: Irritable bowel syndrome
low-FODMAP: Low Fermentable oligo-di-mono-saccharides and polyols
IBS-D: Diarrhoea-predominant irritable bowel syndrome
BMI: Body mass index
HC: Healthy control
IBS-SSS: IBS symptom severity scale
PCoA: Principal co-ordinates analysis
OTU: Operational taxonomy unit
FDR: False discovery rate
LEfSe: Linear discriminant analysis effect size
LDA: Linear discriminant analysis
SAS / SDS: Self-reported Anxiety and Depression Scales
HADS: Hospital Anxiety and Depression Scale
HAM-A / HAM-D: Hamilton Anxiety and Depression Scales
CCA: Canonical correspondence analysis
UHPLC MS/MS: Ultra-high performance liquid chromatography tandem mass spectrometry
OPLS-DA: Orthogonal partial least squares-discrimination analysis
VIP: Variable importance in the projection
KEGG: Kyoto Encyclopedia of Genes and Genomes
FC: Fold change
ANOSIM: Analysis of similarity
SCFAs: Short chain fatty acids
PI-IBS: Post-infectious irritable bowel syndrome
1HNMR: Proton nuclear magnetic resonance
