# Supplementary material for: Microbial and metabolomic profiles in correlation with depression and anxiety co-morbidities in diarrhoea-predominant IBS patients
Source: BMC Microbiol. 2020 Jun 17;20:168. doi: 10.1186/s12866-020-01841-4 (PMC7302156; doi:10.1186/s12866-020-01841-4)
Supplement: Supplementary file 1 — Additional file 1. [file 12866_2020_1841_MOESM1_ESM.docx]

**Additional Material for：**

**Microbial and Metabolomic Profiles in Correlation with Depression and Anxiety Co-morbidities in** **Diarrhoea-predominant IBS Patients**

Tong Liu^1,2,3^, Xiang Gu^1,2,3^, Li-Xiang Li^1,2,3^, Ming Li^1,2,3^,

Bing Li^1,2,3^, Xiao Cui^1,2,3^, Xiu-li Zuo^1,2,3^

^1^Department of Gastroenterology, Qilu Hospital of Shandong University, Jinan, China.
^2^Laboratory of Translational Gastroenterology, Qilu Hospital of Shandong University, Jinan, China

^3^Robot engineering laboratory for precise diagnosis and therapy of GI tumour, Qilu Hospital of Shandong University, Jinan, China

**Corresponding Author:** Dr Xiu-li Zuo, Department of Gastroenterology, Qilu Hospital, Shandong University, 107 Wenhuaxi Road, Jinan 250012, Shandong Province, China; zuoxiuli@sdu.edu.cn; + 86 155 8881 8685

**Figure S1. Taxonomic difference based on linear discriminant analysis (LDA) score.** IBS, IBS-D patients; HC, healthy controls.


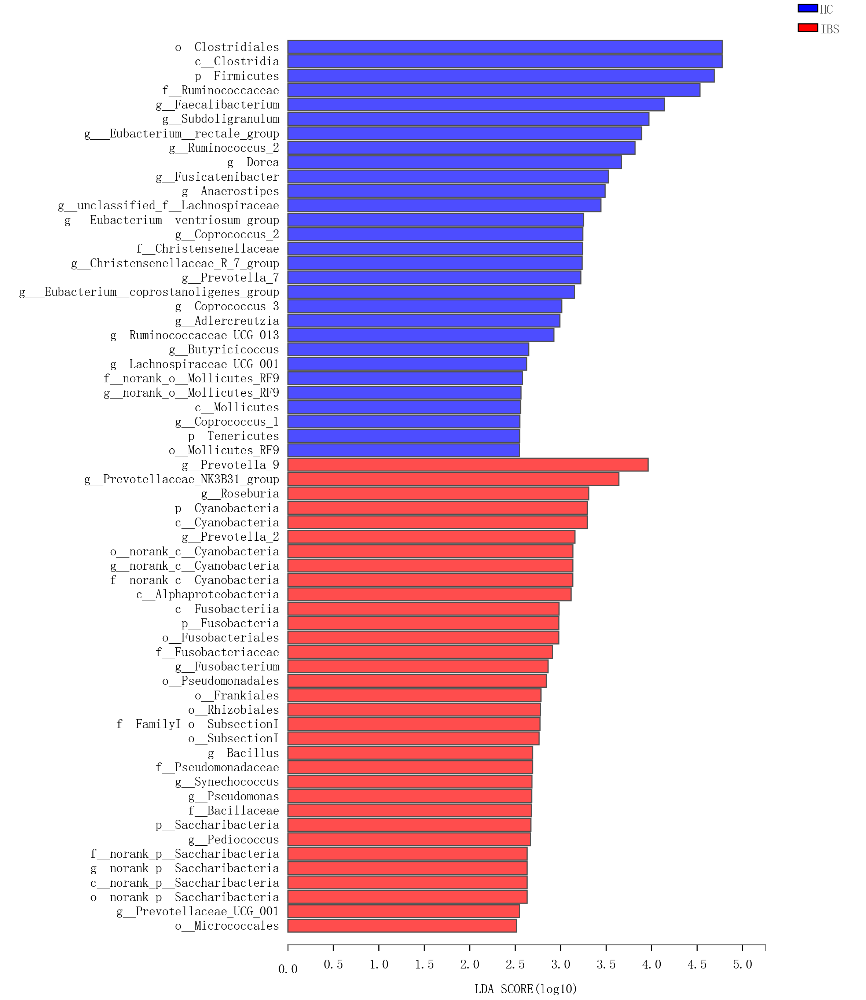


**Figure S2. Correlation network between gut microbial genera in IBS-D and differentiated urinary metabolites harvested in (A) ESI^－^mode and (B) ESI^＋^ mode.** Sizes of the nodes represent for relative abundances of genera (pink) and concentration of metabolites (orange). Red edges indicate Spearman’s correlation coefficient > 0.3 and blue edges indicate Spearman’s correlation coefficient < -0.3. *P* < 0.05.

**
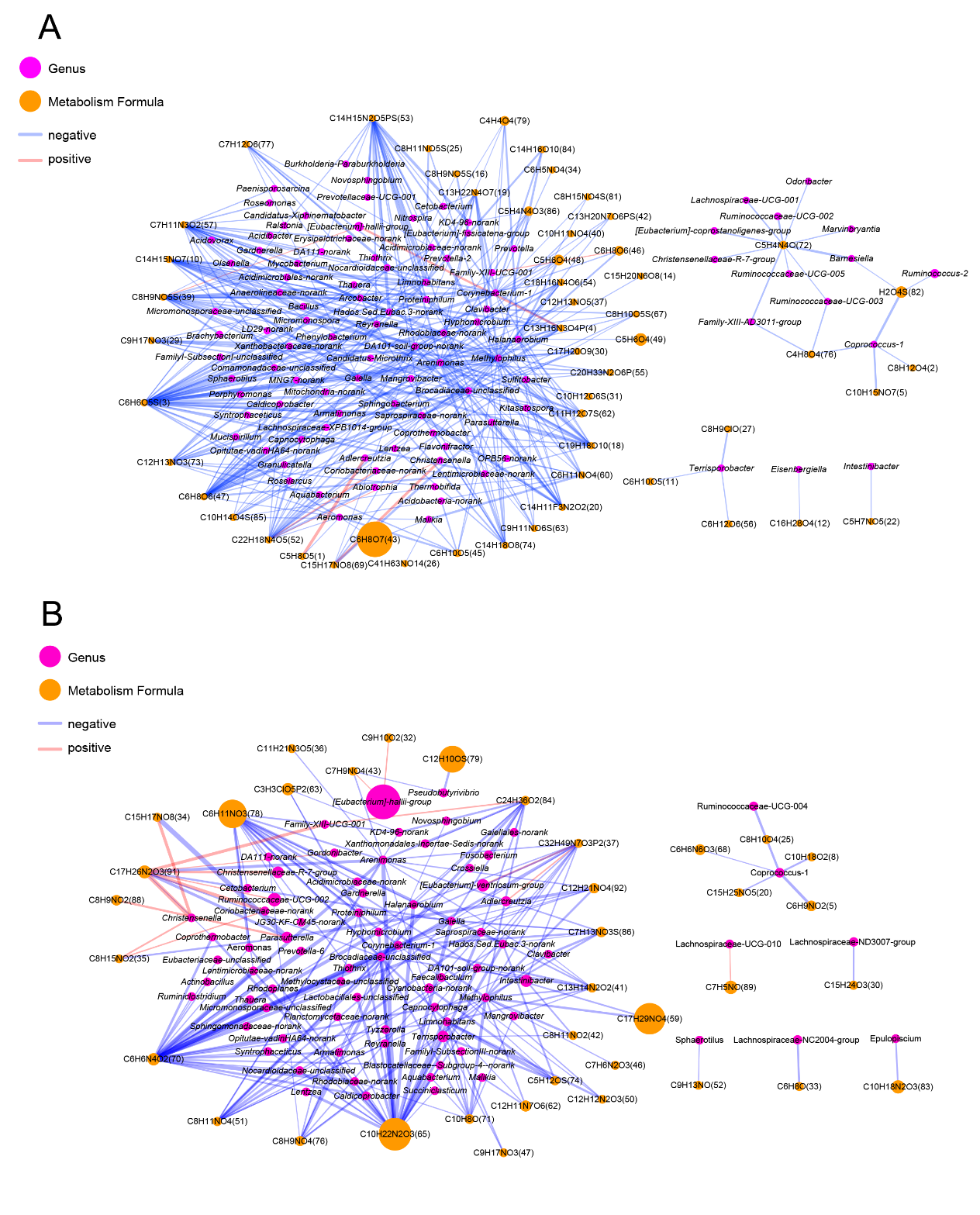
**

**Table S1. Contribution of environmental factors included in CCA ordination via envfit function.**

|  | CCA1 | CCA2 | r^2^ | *P* values |
| --- | --- | --- | --- | --- |
| IBS_SSS | - 0.9842 | 0.1768 | 0.144 | 0.005 |
| SDS | - 0.6525 | - 0.7578 | 0.1826 | 0.002 |
| SAS | - 0.6615 | - 0.7499 | 0.3645 | 0.001 |
| HADS | - 0.9435 | - 0.3314 | 0.207 | 0.001 |
| HAM_D | - 0.9993 | 0.0363 | 0.2824 | 0.001 |
| HAM_A | - 0.9619 | - 0.2732 | 0.2642 | 0.001 |

CCA1, CCA2: projection distance of an environmental factor on CCA axis. r^2^: determination coefficients of an environmental factor upon species community.

**Table S6. Studies on metabolomic changes between IBS and healthy controls.**

| Study | Year | Region | Comparison of Subjects | Material | Method | No. of differential metabolites |
| --- | --- | --- | --- | --- | --- | --- |
| Zeber-Lubecka *et al.* [1] | 2016 | Poland | IBS patients (n = 29) *vs.* healthy volunteers (n = 8) | Faeces | GC/MS | 13 (at baseline) |
| Yu *et al.* [2] | 2018 | China | Water avoidance IBS model mice (n=6) *vs.* HC mice (n=6) | Faeces | GC/MS | 14 |
| Palma *et al.* [3] | 2017 | Canada | Mice transplanted with IBS-D faeces (n=30) *vs.* Mice transplanted with HC faeces (n=22) | Serum | LC/MS | 7 |
| Zhang *et al.* [4] | 2019 | China | IBS-D patients (n=30) *vs.* healthy controls (n=15), age- and sex-matched | Faeces | GC/MS  LC/MS  UPLCA | 6 |
| Shankar *et al.* [5] | 2015 | America | IBS-D children (n=22) vs. healthy children (n=22) | Faeces | ^1^HNMR | 7 |
| Hamid *et al.* [6] | 2018 | Iran | IBS-D patients (n=8) *vs.* healthy volunteers (n=16) | Serum and urine | ^1^HNMR | 5 in serum,  3 in urine |
| Lee *et al*. [7] | 2020 | Korea | IBS-D patients (n = 29) vs. healthy (n = 22) | Faeces | ^1^HNMR | 34 |
| Zhu *et al.* [8] | 2019 | China | IBS-D patients (n=15) *vs.* healthy controls (n=15) | Faeces | GC-TOFMS | 31 |
| Yamamoto *et al.* [9] | 2019 | Canada | IBS patients time point 1 (n = 42) *vs.* IBS patients time point 2 (n = 34) *vs.* healthy volunteers (n = 20) | Urine | MSI-CE-MS | 10 |
| Our study | - | China | IBS-D patients (n=78) *vs.* healthy volunteers (n=42) | Urine | UHPLC-MS/MS | 114 |

GC/MS: Gas chromatography / mass spectrometry. LC/MS: liquid chromatography / mass spectrometry. ^1^HNMR: proton nuclear magnetic resonance. MSI-CE-MS: multi-segment injection-capillary electrophoresis–mass spectrometry. GC-TOFMS: gas chromatography coupled to time-of-flight mass
spectrometry. UHPLC-MS/MS: ultra-high performance liquid chromatography / tandem mass spectrometer. IBS, irritable bowel syndrome. IBS-D, diarrhoea-predominant IBS. HC, healthy controls.

**Supplementary references**

1. Zeber-Lubecka N, Kulecka M, Ambrozkiewicz F, Paziewska A, Goryca K, Karczmarski J, et al. Limited prolonged effects of rifaximin treatment on irritable bowel syndrome-related differences in the fecal microbiome and metabolome. Gut Microbes. 2016;7:397–413.

2. Yu LM, Zhao KJ, Wang SS, Wang X, Lu B. Gas chromatography/mass spectrometry based metabolomic study in a murine model of irritable bowel syndrome. World J Gastroenterol. 2018;24:894–904.

3. Palma G De, Lynch MDJ, Lu J, Dang VT, Deng Y, Jury J, et al. Transplantation of fecal microbiota from patients with irritable bowel syndrome alters gut function and behavior in recipient mice. Sci Transl Med. 2017;9:eaaf6397.

4. Zhang WX, Zhang Y, Qin G, Li KM, Wei W, Li SY, et al. Altered profiles of fecal metabolites correlate with visceral hypersensitivity and may contribute to symptom severity of diarrhea-predominant irritable bowel syndrome. World J Gastroenterol. 2019;25:6416–29.

5. Shankar V, Homer D, Rigsbee L, Khamis HJ, Michail S, Raymer M, et al. The networks of human gut microbe-metabolite associations are different between health and irritable bowel syndrome. ISME J. 2015;9:1899–903. doi:10.1038/ismej.2014.258.

6. Noorbakhsh H, Yavarmanesh M, Mortazavi SA, Adibi P, Moazzami AA. Metabolomics analysis revealed metabolic changes in patients with diarrhea-predominant irritable bowel syndrome and metabolic responses to a synbiotic yogurt intervention. Eur J Nutr. 2018;:1–11.

7. Lee JS, Kim SY, Chun YS, Chun YJ, Shin SY, Choi CH, et al. Characteristics of fecal metabolic profiles in patients with irritable bowel syndrome with predominant diarrhea investigated using 1H-NMR coupled with multivariate statistical analysis. Neurogastroenterol Motil. 2020; December 2019:1–13.

8. Zhu S, Liu S, Li H, Zhang Z, Zhang Q, Chen L, et al. Identification of Gut Microbiota and Metabolites Signature in Patients With Irritable Bowel Syndrome. Front Cell Infect Microbiol. 2019;9 October:1–12.

9. Yamamoto M, Ines M, Sanchez P, Bercik P, Britz P. Metabolomics reveals elevated urinary excretion of collagen degradation and epithelial cell turnover products in irritable bowel syndrome patients. Metabolomics. 2019;15:1–18. doi:10.1007/s11306-019-1543-0.
